# The prognostic utility of HDL as a biomarker for sepsis or septic shock: A systematic review and meta-analysis

**DOI:** 10.1097/MD.0000000000049261

**Published:** 2026-06-12

**Authors:** Yuxia Wang, Fang Han, Lulu Lou, Shubao Wang, Changgen Yang

**Affiliations:** a Department of Endocrinology, Zhejiang Provincial People’s Hospital, Affiliated People’s Hospital, Hangzhou Medical College, Hangzhou, People’s Republic of China; b Department of Intensive Care Unit, Emergency and Intensive Care Unit Center, Zhejiang Provincial People’s Hospital, Affiliated People’s Hospital, Hangzhou Medical College, Hangzhou, People’s Republic of China.

**Keywords:** HDL, Lipoproteins, Mortality, Sepsis

## Abstract

**Background:**

We systematically reviewed and meta-analyzed prospective observational evidence on the association between circulating high-density lipoprotein (HDL) levels and short-term mortality in adult patients with sepsis or septic shock.

**Methods:**

The databases of PubMed, EMBASE, Cochrane Library, CBM, CNKI, Wanfang and VIP were searched for all the studies. RevMan5.4 was used to analyze the sensitivity and heterogeneity and then calculate the combined effect. Funnel plots were applied to evaluate the publication bias of the included studies.

**Results:**

A total of 9 studies were included and synthesized in the analysis. 867 patients were divided into the survival group (*n* = 562) and the death group (*n* = 305). The results showed that the HDL levels increased in sepsis survivors compared to non-survivors (standardized mean difference = 4.67, 95% confidence interval = 1.01–8.34, *Z* = 2.50, *P* = .01, *I*^2^ = 52%). There were higher levels of low-density lipoprotein (LDL) and cholesterol among patients in the survival group than among those in the death group. The triglycerides levels had no significant difference between the 2 groups. Sensitivity analysis indicated that the results were stable. The funnel plot showed that the effect points of the 9 studies were roughly in the form of “inverted funnels.”

**Conclusion:**

Lower HDL levels measured early in sepsis are associated with higher short-term mortality, but the current evidence is observational and of low certainty. HDL may be useful as an adjunctive risk stratification marker rather than a stand-alone decision tool, and its therapeutic relevance requires prospective validation.

## 1. Introduction

Sepsis is a life-threatening organ dysfunction caused by a dysregulated host response to infection, which is associated with high mortality worldwide. According to World Health Organization statistics, the incidence of sepsis among hospitalized patients is 29.5%, increasing to 47% in intensive care units. The mortality rate of sepsis ranges from 33% to 35% and is reportedly increasing by 9% to 13% annually.^[1–6]^ Moreover, the mortality rate can reach 40% to 50% in patients with septic shock.^[7,8]^ Given the high mortality associated with sepsis, the Surviving Sepsis Campaign has emphasized the importance of early screening and timely intervention for patients at high risk.^[9]^ Therefore, reliable biomarkers are valuable for the early diagnosis and treatment of sepsis, thereby improving prognosis and reducing medical costs. Biomarkers are important indicators with the potential to facilitate early disease diagnosis and prognostic assessment. Currently, more than 170 biomarkers have been associated with the pathogenesis of sepsis, such as C-reactive protein and procalcitonin, which have been identified as useful markers for diagnosing sepsis.^[10]^ However, there are few prognostic indicators for assessment in patients with septic shock.

It is well established that HDL levels are negatively correlated with the risk of coronary heart disease. Low-density lipoprotein cholesterol (LDL-C) particles can deposit in the intima of the vascular wall through injured endothelial cells, attracting inflammatory cells that attempt to phagocytose and eliminate them, thereby contributing to the formation of atherosclerotic plaques. In contrast, high-density lipoprotein cholesterol (HDL-C) exerts protective vascular effects by transporting cholesterol (TC) to the liver and adrenal glands and promoting the clearance of LDL-C through the intestinal tract. In addition, HDL-C has antioxidant and anti-inflammatory properties, which help prevent LDL-C oxidation and inflammatory cell migration.^[11]^

Recently, an increasing number of clinical studies have confirmed the association between HDL levels and mortality in patients with sepsis or septic shock. However, the findings remain inconclusive. In addition, most of these studies have been limited by relatively small sample sizes, and their results have not been entirely consistent. To date, no meta-analysis has comprehensively evaluated the predictive value of HDL in the development and prognosis of sepsis. Therefore, this systematic review and meta-analysis aim to synthesize the existing evidence and assess the utility of HDL as a prognostic biomarker in patients with sepsis or septic shock, thereby providing evidence to support clinical diagnosis and treatment.

## 2. Methods

### 2.1. Protocol/registration

This study was performed in accordance with the 2020 Preferred Reporting Items for Systematic Reviews and Meta-Analyses (PRISMA) statement.^[12]^ The systematic review was registered in PROSPERO (CRD42022337934). This study was performed in accordance with the 2020 PRISMA statement.^[12]^ The systematic review was registered in PROSPERO (CRD42022337934). Since the identities and information of all patients were disclosed in the original study, the patient-related information is open and transparent, and there is no need to involve ethics, and our study did not compromise the privacy of patients.

### 2.2. Information source and search strategy

We searched PubMed, Cochrane Library, Embase, China Wan fang Database, China National Knowledge Infrastructure and Biomedical Literature Database of China from their date of inception to December 31, 2023, and then completed an updated literature on March 01, 2024, to include any more recent studies. The Me SH terms and keywords are sepsis/septic shock and lipoprotein/HDL/LDL/TC/triglycerides (TG). Meanwhile, the citations of included studies were screened to identify additional studies for inclusion.

**Criteria for included studies:** The patients should be adults (>18 years of age) with sepsis (severe sepsis or septic shock); the mortalities were clearly shown in the results of the studies; the studies should be Prospective observational studies or randomized controlled trials, limited to English and Chinese; studies with insufficient data should be excluded. and patients with liver disease (liver cirrhosis, immune hepatitis, hepatitis B, and hepatocellular carcinoma), dyslipidemia, or a history of steroid use within the previous 7 days were excluded.

### 2.3. Data extraction and management

According to the predefined criteria, 2 reviewers independently screened the studies through a 2-step process: first by reviewing the titles and abstracts, and then by assessing the full texts. Any disagreements regarding study eligibility were resolved through discussion or, when necessary, consultation with a third reviewer. If required, the authors of the original studies were contacted to obtain additional information. The extracted data included the following: bibliographic details, including the first author, publication year, study setting, and country; clinical characteristics, including sample size, mean age, and study type (prognostic or diagnostic); and study outcomes, including HDL, LDL, TG, and TC levels, as well as mortality proportion.

### 2.4. Evaluation of literature quality

Referring to the quality assessment scales of nonexperimental studies,^[13,14]^ the Newcastle-Ottawa scale (NOS) was used to evaluate the quality of the literature. The NOS allocates a maximum of 9 points according to the quality of the selection, comparability, and outcomes of the cohort study populations. Accordingly, all of the non-randomized studies included in the meta-analyses were classified as low-risk of bias (6–9 points), intermediate-risk (4–5 points) or high risk (1–3 points).

### 2.5. Statistical analysis

All statistical analyses were performed using RevMan V5.4. First, the *χ*2 test and *I*^2^ test were adopted to evaluate the heterogeneity between studies. If there was no heterogeneity between studies (*I*^2^ < 50%), a fixed effect model was used; Otherwise, a random effect model was used to identify possible causes of heterogeneity between studies and calculate the combined effect after excluding. The measurement data included in this study were presented as standardized mean difference (SMD) and its 95% confidence interval (95% CI), and the funnel plot was used to analyze publication bias in the studies. If the study reported mean error and standard error, the SD was calculated using the formula provided by the Cochrane Collaboration.^[15]^ If the mean biomarker data were not reported, the mean and SD were estimated according to the median and interquartile range.^[16]^ A *P* value <0.05 (*P* < .05) was considered significant.

## 3. Result

### 3.1. Study selection

**Figure 1.**
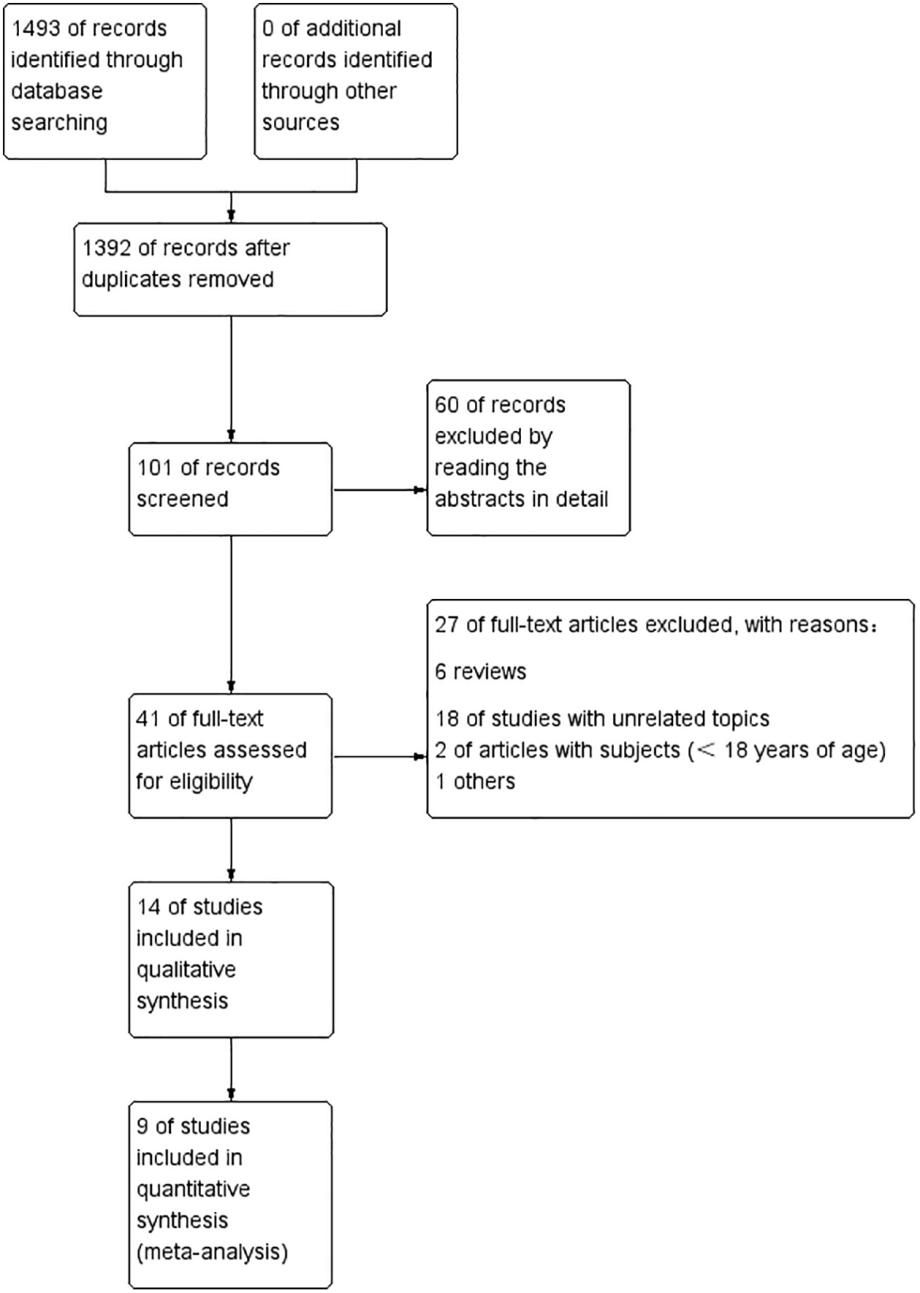
Flowchart of literature selection.

A total of 1493 records were identified. After removal of 1392 duplicates, 101 records remained for title/abstract screening, of which 60 were excluded. Forty-one full-text articles were reviewed in detail; 27 were excluded for not meeting the eligibility criteria, and 5 were excluded because extractable quantitative data were unavailable. Nine studies were finally included. NOS scores ranged from 6 to 8, suggesting moderate-to-good methodological quality, although several studies still carried important risks of confounding and exposure-timing bias. The selection process is shown in Figure 1.

### 3.2. Study/patient characteristics

Nine prospective observational studies with a total of 867 patients were included.^[17–25]^ Baseline characteristics and available results included in the studies are shown in Table 1. All 9 studies were prospective observational and published in English. The mean age of the patients ranged from 56 to 71.2 years old.^[21,24]^ Most of the studies did not investigate the source of infection, but some studies^[18,19,23]^ showed that lungs, abdomen, and urinary tract were the most common sources of infection. The mortality rates of each study ranged widely, from 12.98% to 53.6%.^[23–25]^

**Table 1:** The basic characteristics of studies included in meta-analysis.

Study author	Yr	Country	Ages	Study setting	Object of study	N	Data provided	Ending
Stefan^[18]^	2008	Germany	59 ± 26	ICU	Sepsis or sepsis shock	151	HDL and LDL	Death
Ber bée JF^[17]^	2008	Netherlands	65 ± 20	ICU	Sepsis or sepsis shock	17	HDL and LDL	Death
Lekker A^[19]^	2014	Skinner	63.7 ± 3.5	Ward	Sepsis	50	HDL and LDL	Death
Lee SH^[20]^	2015	Korea	62.7 ± 16.2	ICU	Sepsis or sepsis shock	117	HDL and LDL	Death
Faheem W^[22]^	2017	America	64 ± 14	ED	Sepsis	35	HDL and LDL	Death
Cirstea M^[21]^	2017	Canada	56	ED	sepsis	200	HDL and LDL	MODS or Death
Guirgis FW^[23]^	2018	America	64 ± 14	ED	sepsis or sepsis shock	97	HDL and LDL	Death
Irfan Karahan^[24]^	2020	Turkey	71.2 ± 9.35	Ward	sepsis or sepsis shock	69	HDL and LDL	Death
Faheem W^[25]^	2021	America	61 ± 19	ED	sepsis or sepsis shock	131	HDL and LDL	Death

ED = emergency department, HDL = high-density lipoprotein, ICU = intensive care unit.

### 3.3. Primary outcome

**Figure 2.**
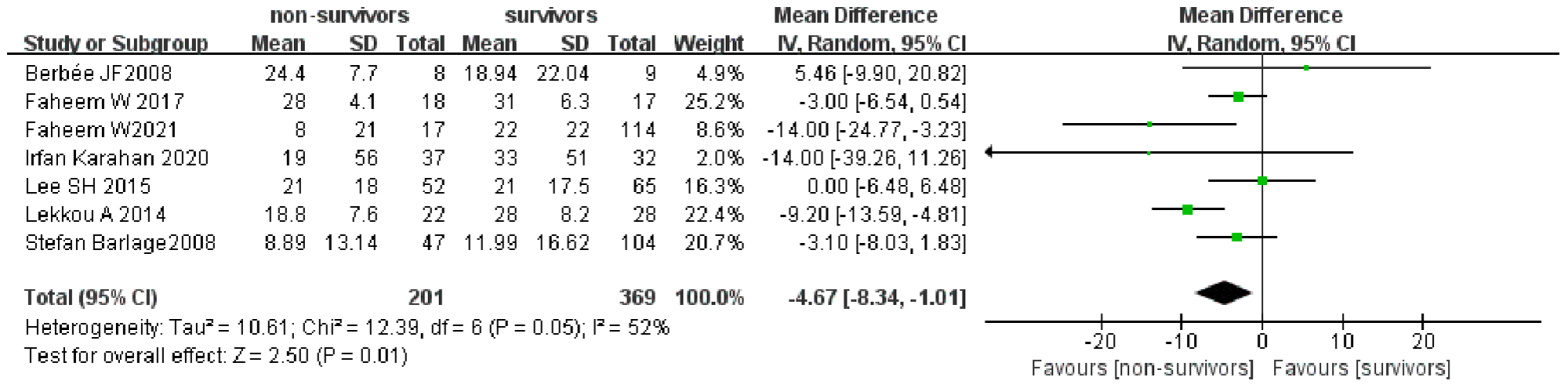
Forest plot for HDL levels. CI = confidence interval, HDL = high-density lipoprotein, SD = standard deviation.

**Figure 3.**
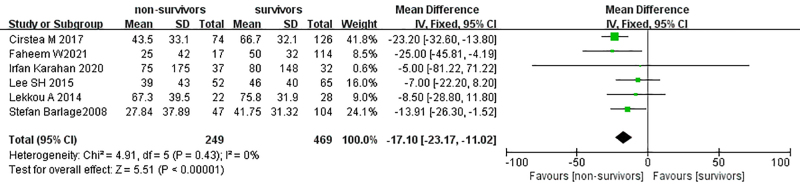
Forest plot for LDL levels. CI = confidence interval, LDL = low-density lipoprotein, SD = standard deviation.

**Figure 4.**
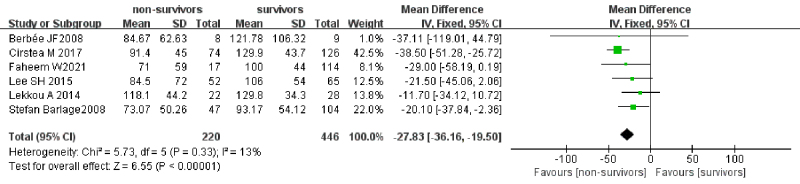
Forest plot for TC levels. CI = confidence interval, SD = standard deviation, TC = cholesterol.

**Figure 5.**
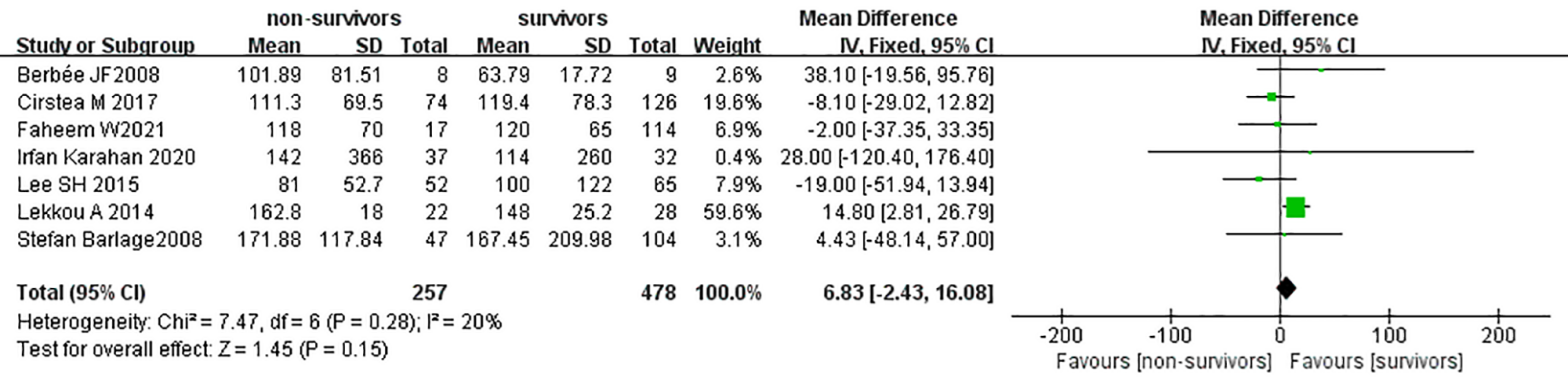
Forest plot for TG levels. CI = confidence interval, SD = standard deviation, TG = triglycerides.

The 9 trials involved were combined for meta-analysis, involving 867 patients, 562 of whom were in the survival group and 305 in the death group. 9 studies reported short-term mortality, of which 3 were in-hospital mortality, 5 were 28-day mortality, and 1 was 30-day mortality. Five studies were conducted in the ICU, 3 in the emergency department and the rest in the general ward. There was no difference in sex, age, platelet count, statins usage and comorbidities between the two groups. The results showed that mortality in patients with sepsis/septic shock was associated with lower HDL levels SMD = 4.67, 95% confidence interval (95% CI) = 1.01~8.34, *Z* = 2.50, *P* = .01, *I*^2^ = 52%] (Fig. 2). There were higher levels of LDL and TC among patients in the survival group than among those in the death group (SMD = −17.1, 95% CI = −23.17–11.02, *Z* = 5.51, *P* < .00001, *I*^2^ = 0% and SMD = −27.73, 95% CI = −36.11–19.36, *Z* = 6.55, *P* < .00001, *I*^2^ = 13%, respectively) (Figure 3, Figure 4). And there was no statistical difference in TG levels (SMD = 6.83, 95%CI = −2.43~16.08, *Z* = 1.45, *P* = .15, *I*^2^ = 20%) (Fig. 5).

### 3.4. AUC analysis of HDL as a diagnostic predictor for sepsis/ septic shock

Four studies reported ROC analyses for HDL-related prognostic performance, with AUC values ranging from 0.600 to 0.818. This broad range suggests only modest-to-good discrimination and indicates that HDL should not be interpreted as a uniformly strong stand-alone predictor. Only 1 study reported sensitivity and specificity explicitly (sensitivity 85.7%, specificity 69.9%), whereas the other studies reported only AUCs and/or odds ratios, limiting quantitative synthesis of diagnostic performance. Accordingly, the ROC evidence should be interpreted as hypothesis-generating rather than practice-changing (Table 2).

**Table 2:** ROC analyses for prediction of sepsis-related mortality according to baseline HDL levels.

Study author	Ending	AUC	Cutoff (mg/dL)	OR	95% CI	Sn	Sp	PPV	NPV
Cirstea M^[21]^	28-d	0.818	25.1	–	–	0.857	0.699	0.176	0.985
Irfan Karahan^[24]^	Mortality	–	32	1.85	0.68–4.99	–	–	–	–
Lekker A^[19]^	Mortality	–	–	1.3	1.03–1.63	–	–	–	–
Stefan^[18]^	30-d	0.6	–	–	–	–	–	–	–

AUC = area under curve, HDL = high-density lipoprotein, NPV = negative predictive value, PPV = positive predictive value, Sn = sensitivity, Sp = specificity.

### 3.5. Sensitivity analysis

Leave-one-out sensitivity analysis showed that the direction of the HDL association remained unchanged after exclusion of each study. The pooled MD ranged from −5.58 to −3.20 mg/dL across iterations, and heterogeneity ranged from 25.7% to 58.2%. Omission of the Lekki study attenuated the pooled effect most strongly and reduced heterogeneity, suggesting that differences in case mix, disease severity, or lipid sampling may partly explain the moderate between-study heterogeneity.

### 3.6. Publication bias

Visual inspection of the HDL funnel plot suggested approximate symmetry, but interpretation is limited by the small number of studies. Egger’s regression test did not indicate statistically significant small-study effects (*P* = .846). Nevertheless, publication bias cannot be excluded, and selective reporting remains possible because several studies did not provide complete diagnostic performance metrics or full adjustment models (Table 3).

**Table 3:** Exploratory subgroup and meta-regression analyses for HDL.

Analysis	Studies	Pooled MD (mg/dL)	95% CI	I^2^	*P*-value
ICU subgroup	3	−1.51	−5.31 to 2.29	0.0%	
ED subgroup	2	−7.28	−17.79 to 3.23	72.4%	
Ward subgroup	2	−9.34	−13.67 to −5.01	0.0%	
Subgroup difference	7	–	–	–	.027
Egger’s test	7	–	–	–	.846
Meta-regression (yr/ setting)	7	–	–	–	Yr 0.268; setting 0.229

ED = emergency department, HDL = high-density lipoprotein, ICU = intensive care unit, MD = mean difference.

Meta-regression results are exploratory because only seven HDL datasets were available.

**Figure 6.**
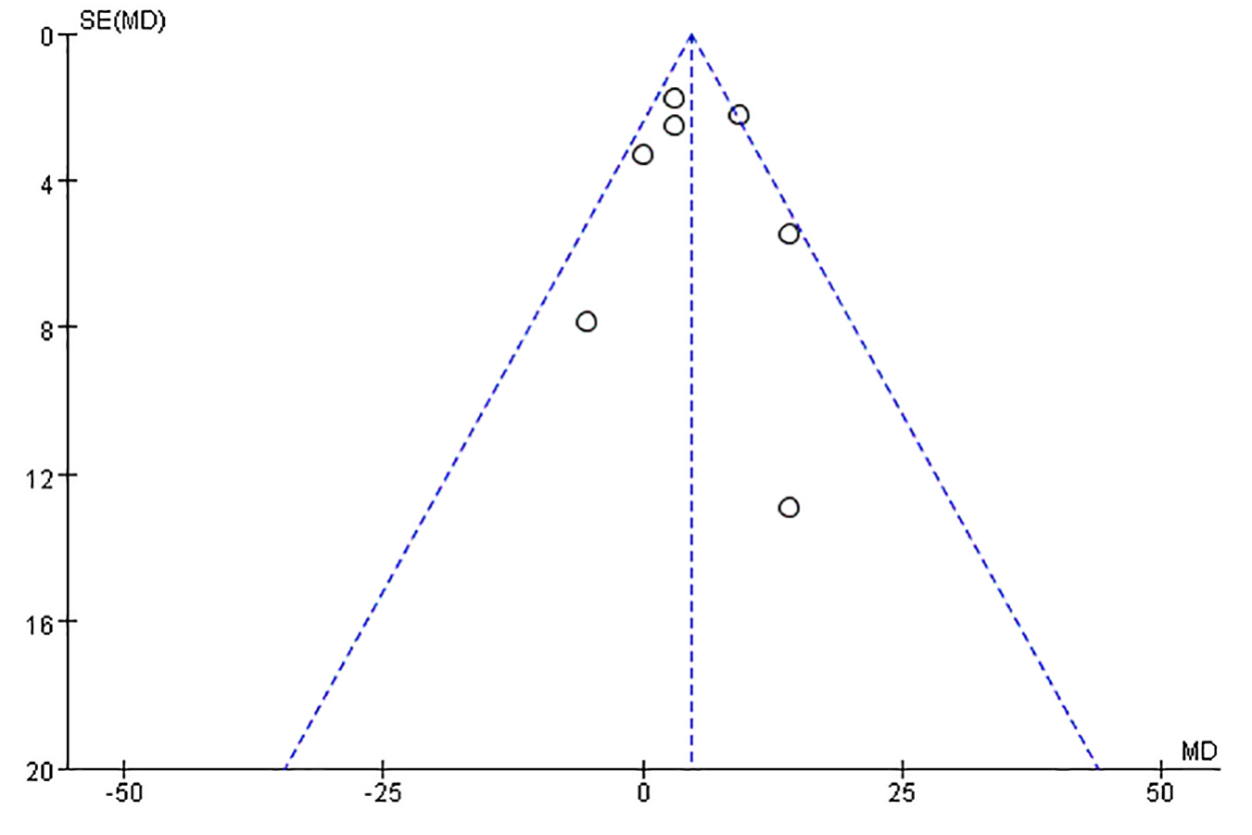
Funnel plot for HDL levels. HDL = high-density lipoprotein, MD = mean difference, SD = standard deviation.

A funnel plot about HDL and mortality in patients with sepsis showed that the effect points of the studies were in the form of an “inverted funnel” with a large symmetric distribution centered on the combined effect (Fig. 6). The number of studies included in the meta-analysis is relatively small, and publication bias cannot be completely ruled out (Table S1, Supplemental Digital Content 1).

## 4. Discussion

Sepsis is a life-threatening disease remaining a global health concern, which is difficult to predict and treatment.^[26,27]^ Apart from early antibiotic application and supportive care, there is no specific treatment for sepsis. It’s important to determine the infected patients who are likely to develop sepsis. These patients can be targeted for early intensive surveillance. It was recognized that the levels of TC, HDL and LDL are reduced in patients with sepsis.^[28–30]^ Levels are decreased at the time of diagnosis and often decline further during the disease course. The levels of HDL were at their lowest around day 3 post-admission, and the LDL levels at the time of diagnosis. However, variable recovery in serum levels occurs in the following days along with compensation and timely treatment.^[20]^ Reasons for decreased HDL, LDL and TC levels in sepsis may include decreased hepatic synthesis, impaired TC transport, increased metabolism, provision of substrate for steroid biosynthesis and depletion through toxin scavenging.^[31–33]^

This study is the first comprehensive meta-analysis to evaluate the prognostic value of HDL as a biomarker in sepsis. Only prospective observational studies were included to minimize potential bias. The meta-analysis demonstrated significant differences in lipoprotein levels between survivors and non-survivors within 24 hours of sepsis diagnosis. Specifically, HDL, LDL, and TC levels were significantly higher in the survivor group than in the non-survivor group. However, no significant difference in triglyceride levels was observed between the 2 groups. Several mechanisms may explain the protective role of HDL-C in sepsis, including binding to and facilitating the clearance of bacterial toxins, suppressing monocyte activation, modulating macrophage and dendritic cell function, reducing the release of inflammatory cytokines, and inhibiting the expression of vascular and intercellular adhesion molecules.^[34]^ All of these processes will reduce inflammatory response and protect the body from damage. Once patients were diagnosed with sepsis, the levels of HDL, LDL and TC in them would decrease, which can lead to organ damage or even death.^[35–37]^ The low levels of HDL and LDL have also been associated with poor sepsis outcomes. Karahan found that an HDL-C level lower than 25.1 mg/dL at admission was highly prognostic for every adverse outcome examined.

Recent studies have also deepened the biological interpretation of HDL in sepsis. A 2024 cross-sectional study showed that the anti-inflammatory capacity of apo-depleted plasma was reduced in septic patients, correlated with the SOFA score and inflammatory markers, and appeared to be closely linked to HDL concentration.56 A 2025 prospective study further suggested that combining an HDL subfraction (HDL2b) with SOFA improved discrimination for short-term mortality compared with SOFA alone.58 These findings support the concept that HDL biology in sepsis involves both quantity and function. However, they also imply that simple HDL-C measurement captures only part of the pathophysiological signal (Table S2, Supplemental Digital Content 2).

Many experimental studies have shown that HDL exerts multiple biological effects, including anti-inflammatory, antiapoptotic, and antioxidant properties, and can also bind to and neutralize lipopolysaccharide. Therefore, HDL-based strategies, such as recombinant HDL (rHDL) and PCSK9-targeted interventions, have been explored as potential therapeutic approaches in patients with sepsis. Pajkrt et al investigated the effects of rHDL in human endotoxemia and found that rHDL attenuated the endotoxin-induced inflammatory response by alleviating clinical symptoms, such as chills, myalgia, backache, and vomiting, and by markedly reducing the release of proinflammatory cytokines, including tumor necrosis factor-α, interleukin-6, interleukin-8. It is well known that sepsis is frequently accompanied by coagulation disorders. Notably, rHDL infusion can influence fibrinolytic activity and directly affect platelet function by reducing platelet aggregation, thereby modifying the procoagulant state associated with endotoxemia. This suggests that the procoagulant effects associated with sepsis may be better controlled through rHDL-based interventions. Levels et al further demonstrated that prophylactic administration of rHDL induced changes in lipid metabolism in healthy subjects exposed to endotoxin. In addition to findings similar to those of previous studies regarding cytokines and procoagulant factors, they observed increased levels of TC, apolipoprotein A-I, and HDL in the group pretreated with rHDL, suggesting that rHDL may increase phospholipid levels, which are important components of VLDL, LDL, and HDL. However, some animal studies have reported adverse effects associated with rHDL administration, including elevated liver transaminase levels and epileptic seizures, and have failed to demonstrate an improvement in prognosis. In addition, PCSK9 inhibitors prevent LDL receptor degradation by binding to PCSK9 and are typically used in patients with refractory hyperlipidemia, where they can substantially reduce LDL-C levels. Some studies have suggested that PCSK9 inhibitors and statins may be beneficial in the treatment of sepsis, whereas others have not supported their use in septic patients. Therefore, prospective randomized controlled trials are still needed to clarify their potential therapeutic effects, particularly in specific patient subgroups.

In conclusion, lower early HDL levels are associated with higher short-term mortality in patients with sepsis or septic shock, and similar directional associations are seen for LDL and TC. However, because the available evidence is observational, heterogeneous, and of low certainty, these findings should be interpreted as supportive of prognostic enrichment rather than as a basis for immediate practice change. HDL may be incorporated into future multimarker models and mechanistic studies, but it cannot yet be recommended as a stand-alone clinical decision tool or therapeutic target.

### 4.1. Limitations

This review has several limitations. First, the number of studies contributing quantitative HDL data was small, which reduces power for publication bias testing and meta-regression. Second, all included studies were observational, so residual confounding by illness severity, nutritional status, lipid-lowering therapy, infection source, and comorbidity burden cannot be excluded. Third, study settings and mortality endpoints varied across reports, and lipid sampling was not perfectly standardized, which likely contributed to the moderate heterogeneity observed for HDL. Fourth, baseline pre-sepsis lipid levels were unavailable, so the extent to which low HDL reflects chronic metabolic phenotype versus acute inflammatory consumption could not be determined. Fifth, diagnostic performance reporting was incomplete: only 1 study provided both sensitivity and specificity, preventing robust pooled ROC synthesis. Finally, although PRISMA reporting was updated during revision, the evidence base itself remains limited by small single-center cohorts and heterogeneous adjustment strategies.

### 4.2. Future directions

Future studies should prospectively standardize the timing of lipid sampling, prespecify short-term mortality endpoints, and report complete diagnostic performance metrics together with calibration and incremental value over established sepsis scores. Multicenter cohorts should evaluate whether HDL adds clinically relevant discrimination beyond SOFA, lactate, and other routinely available variables. Mechanistic studies should distinguish HDL quantity from HDL functionality and HDL subfractions, while interventional research should proceed cautiously and only after stronger causal evidence has been established. At present, the most clinically defensible role for HDL is as a candidate adjunctive biomarker for early risk stratification and enrichment of future sepsis trials.

## Author contributions

**Conceptualization:** Yuxia Wang, Changgen Yang.

**Data curation:** Yuxia Wang, Changgen Yang.

**Formal analysis:** Yuxia Wang, Lulu Lou, Shubao Wang, Changgen Yang.

**Methodology:** Yuxia Wang, Fang Han, Lulu Lou, Shubao Wang, Changgen Yang.

**Project administration:** Changgen Yang.

**Supervision:** Changgen Yang, Fang Han, Shubao Wang.

**Validation:** Fang Han, Changgen Yang.

**Visualization:** Yuxia Wang, Fang Han, Changgen Yang.

**Investigation:** Fang Han, Lulu Lou, Changgen Yang.

**Resources:** Changgen Yang.

**Software:** Changgen Yang.

**Writing – original draft:** Yuxia Wang.

**Writing – review & editing:** Changgen Yang.




