# Supplementary material for: The prognostic utility of HDL as a biomarker for sepsis or septic shock: A systematic review and meta-analysis
Source: Medicine (Baltimore). 2026 Jun 12;105(24):e49261. doi: 10.1097/MD.0000000000049261 (PMC13268517; doi:10.1097/MD.0000000000049261)
Supplement: Supplementary file 1 [file medi-105-e49261-s001.docx]

Table S1 Domain-based risk-of-bias considerations for included studies

| **Study** | **Selection bias** | **Confounding** | **Exposure timing / measurement** | **Overall concern** |
| --- | --- | --- | --- | --- |
| Berbée 2008 | Moderate | Moderate | Moderate | Moderate |
| Barlage 2009 | Moderate | Moderate | Low to moderate | Moderate |
| Lekkou 2014 | Moderate | Moderate | Moderate | Moderate |
| Lee 2015 | Moderate | Moderate | Low | Moderate |
| Guirgis 2017 | Moderate to high | High | Low to moderate | High |
| Cirstea 2017 | Moderate | Moderate | Low | Moderate |
| Guirgis 2018 | Moderate | High | Low to moderate | High |
| Karahan 2020 | Moderate | High | Low | High |
| Guirgis 2021 | Moderate | Moderate to high | Low to moderate | Moderate to high |
