# Supplementary material for: The prognostic utility of HDL as a biomarker for sepsis or septic shock: A systematic review and meta-analysis
Source: Medicine (Baltimore). 2026 Jun 12;105(24):e49261. doi: 10.1097/MD.0000000000049261 (PMC13268517; doi:10.1097/MD.0000000000049261)
Supplement: Supplementary file 2 [file medi-105-e49261-s002.docx]

Table S2 GRADE certainty assessment for the primary HDL outcome

| Outcome | Study design | Risk of bias | Inconsistency | Indirectness | Imprecision | Certainty |
| --- | --- | --- | --- | --- | --- | --- |
| Early HDL level and short-term mortality in sepsis | Observational | Serious | Serious | Not serious | Serious | Low |
